# Lyophilized Lipid Liquid Crystalline Nanoparticles as an Antimicrobial Delivery System

**DOI:** 10.3390/antibiotics12091405

**Published:** 2023-09-04

**Authors:** Muhammed Awad, Timothy J. Barnes, Clive A. Prestidge

**Affiliations:** 1 Centre for Pharmaceutical Innovation, Clinical and Health Sciences, University of South Australia, Adelaide 5000, Australia; muhammed.awad@mymail.unisa.edu (M.A.); tim.barnes@unisa.edu.au (T.J.B.); 2 Basil Hetzel Institute for Translational Health Research, Woodville South 5011, Australia; 3 Pharmaceutical Analytical Chemistry, Faculty of Pharmacy, Azhar University, Assiut 71524, Egypt

**Keywords:** antimicrobial, lyophilization, photodynamic therapy, lipid liquid crystalline, nanoparticles

## Abstract

Lipid liquid crystalline nanoparticles (LCNPs) are unique nanocarriers that efficiently deliver antimicrobials through biological barriers. Yet, their wide application as an antimicrobial delivery system is hindered by their poor stability in aqueous dispersions. The production of dried LCNP powder via lyophilization is a promising approach to promote the stability of LCNPs. However, the impact of the process on the functionality of the loaded hydrophobic cargoes has not been reported yet. Herein, we investigated the potential of lyophilization to produce dispersible dry LCNPs loaded with a hydrophobic antimicrobial compound, gallium protoporphyrin (GaPP). The effect of lyophilization on the physicochemical characteristics and the antimicrobial activity of rehydrated GaPP-LCNPs was studied. The rehydrated GaPP-LCNPs retained the liquid crystalline structure and were monodisperse (PDI: 0.27 ± 0.02), with no significant change in nanoparticle concentration despite the minor increase in hydrodynamic diameter (193 ± 6.5 compared to 173 ± 4.2 prior to freeze-drying). Most importantly, the efficacy of the loaded GaPP as an antimicrobial agent and a photosensitizer was not affected as similar MIC values were obtained against *S. aureus* (0.125 µg/mL), with a singlet oxygen quantum yield of 0.72. These findings indicate the suitability of lyophilization to produce a dry form of LCNPs and pave the way for future studies to promote the application of LCNPs as an antimicrobial delivery system.

## 1. Introduction

The application of lipid-based nanomaterials as a delivery system for therapeutics is a promising approach that can tackle many clinical challenges and promote the efficacy of different therapeutics [1,2]. One promising class of nanomaterials that have shown great potential as a delivery system are lipid liquid crystalline nanoparticles (LCNPs) [3]. The crystalline structure of LCNPs with mesh-like lipid bilayer separated with inner water channels makes them ideal carriers for both hydrophilic and hydrophobic compounds, where hydrophobic molecules are entrapped in the lipid bilayer and hydrophilic compounds are trapped in the water channels [3]. In addition, LCNPs have been shown to efficiently permeate through biological barriers and promote the efficiency of antimicrobial agents [2,4,5]. Recently, we demonstrated that LCNP fabricated from glycerol monooleate (GMO) could promote the potential of gallium protoporphyrin (GaPP) as an antimicrobial against bacterial biofilms [6,7,8]. GaPP was successfully loaded in the LCNP lipid bilayer, which promoted its antibacterial and photodynamic activities through efficient solubilization and enhancing its delivery into bacterial biofilms [8]. The positive impact of LCNP on the antibacterial activity of GaPP is thought to enable its clinical application as a promising antimicrobial photosensitizer [7].

**Figure 1:**
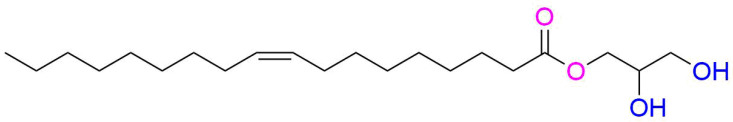
Chemical structure of glycerol monooleate.

However, LCNP colloidal dispersions suffer poor long-term stability and cannot be stored at room temperature for extended periods [9], which limits their practical application. The ester linkage in GMO (Figure 1) is prone to hydrolysis in aqueous solutions [3], which subsequently disrupts the crystalline structure of LCNPs and leads to aggregation of the loaded GaPP in aqueous solutions. Therefore, obtaining LCNPs in a dispersible dry powder is hypothesized to improve the stability and promote the practical application of GaPP-LCNPs [10,11].

Freeze-drying is a simple and more convenient technique to acquire dry powder formulations. Compared to spray drying [11,12,13,14], it can be used for heat-sensitive materials and small-volume samples, and it is easy to scale up [11]. During the freeze-drying process, stress is produced that can disrupt the integrity of nanoparticles; thus, cryoprotective agents are added to protect nanoparticles from freezing stress and improve their stability upon storage [11].

Disaccharides have been successfully used as cryoprotectants for biological samples and nanoformulations [15,16,17,18]. They are thought to protect the lipid bilayer from stress either by replacing water in the spaces between the hydrophilic groups in the lipid bilayer or by forming a protective amorphous matrix around it [19,20]. Trehalose is among the most renowned cryoprotectants that has been widely used in lyophilization of nanoparticles and biological samples [16,21]. The high safety profile and low molecular size of trehalose has prompted its utilization in this study as it is hypothesized it can enter the water channels of LCNPs and provide the necessary protection to the lipid bilayer [22].

A few studies have investigated lyophilization of LCNPs as a tool to promote its stability and shelf-life [9,22,23]. However, there is still a need to elucidate the effect of freeze-drying on the functionality of LCNPs as a delivery system for photodynamic applications. Hydrophobic photosensitizers such as GaPP tend to form dimers via π-π interactions, which lowers their photodynamic activity [1]. Thus, confirming the integrity of LCNPs’ lipid bilayer and the uniform distribution of GaPP in LCNPs after rehydration is essential to validate the use of freeze-drying to obtain dispersible dry GaPP-LCNP formulation. To this end, we investigated the effect of freeze-drying using trehalose as cryoprotectant on the photodynamic activity of GaPP within LCNPs as a proof-of-concept study, which can pave the way for future implementation of GaPP-LCNPs as an antimicrobial agent and a photosensitizer.

## 2. Materials

Glycerol monooleate (Myverol 18–92 K, Kerry ingredients, product number: 4552180, composed of 95% unsaturated monoglycerides) was kindly donated by DKSH Performance Materials Australia. Gallium protoporphyrin was purchased from Frontier Scientific (Logan, UT, USA). Trehalose, Pluronic F127, propylene glycol, uric acid and methanol with HPLC gradient grade ≥ 99.9% were purchased from Sigma Aldrich (St. Louis, MO, USA). Tryptic soy broth (TSB) and agar were purchased from Oxoid Limited; cation-adjusted Muller–Hinton broth was obtained from BD Difco™ (Thermo Fisher Scientific Australia Pty Ltd., Scoresby, VIC, Australia). All the reagents used were of analytical reagent grade, and double-distilled MQ water was used for all experiments conducted.

*Staphylococcus aureus* Xen 29 bioluminescent strain derived from a parental strain from the American Type Culture Collection (ATCC) (part number: 119240) was kindly gifted by Prof. Allison Cowen. *S. aureus*-Xen29 possesses a stable copy of the *Photorhabdus luminescens* lux operon on the bacterial chromosome.

### 2.1. Fabrication of Liquid Crystalline Nanoparticle Dispersions

LCNPs loaded with GaPP were prepared as previously described with slight modifications. Briefly, aqueous dispersion of GaPP-LCNPs was prepared in a scintillation glass by mixing glycerol monooleate (15 mg) with 260 µL of propylene glycol and Pluronic F127 (3 mg) and the methanolic solution of GaPP (1.5 mM). Excess methanol was further added to bring the mixture to a homogenous methanolic solution. Following methanol evaporation under N_2_ gas, the lipid film was reconstituted using an aqueous solution of 2% *w/v* trehalose to obtain a final volume of 5 mL. Blank LCNP samples were prepared similarly by omitting GaPP from the formulation.

### 2.2. Freeze-Drying and Redispersion

LCNP and GaPP-LCNP aqueous dispersions in scintillation glass vials were frozen at −80 °C for 24 h. The frozen samples were lyophilized in a Labconco^®^ Dry Ice bench-top freeze dryer overnight. The dry cakes of LCNPs and GaPP-LCNPs were redispersed with MQ water for characterization.

### 2.3. Determination of Nanoparticle Diameter

The z-average diameter and polydispersity index (PDI) of the nanoparticle dispersions before and after the lyophilization process were determined using Zetasizer Nano ZS (Malvern, Worcestershire, UK). Both LCNP and GaPP-LCNP dispersions were diluted 1:100 in 1 mM KCL, with a refractive index of 1.48, at 25 °C. The data reported were the average of three independent formulations.

### 2.4. Measuring LCNP Concentration

Nanoparticle tracking analysis technique was used to determine the concentrations of blank LCNPs and GaPP-loaded LCNPs before and after lyophilization using Nanosight NS300 (Malvern, Worcestershire, UK) equipped with a blue (405 nm) laser. The samples were diluted 1:100 in Milli-Q water and measured in triplicates at room temperature. The particle motion was recorded using an sCMOS camera, and the data were analyzed using an analysis software (NTA 3.4 Build 3.4.003).

### 2.5. Quantifying GaPP in LCNPs

The concentration of entrapped GaPP within LCNPs before and after the lyophilization process was determined using spectrofluorimetric assay as previously described [7]. The fluorescent signal of GaPP at 585 nm after excitation at 405 nm was examined to determine the concentration of GaPP using a Fluostar^®^ Omega microplate reader. A calibration curve was plotted in the range between 0.3 and 3 µM with a correlation coefficient of 0.9998. GaPP-LCNP dispersions were centrifuged for 10 min at 31,120× *g* to separate unentrapped GaPP from GaPP-loaded LCNPs. The supernatant containing GaPP-LCNPs was collected and dissolved in methanol to release GaPP from LCNPs. Aliquots of the released GaPP was further diluted in methanol and the concentration was determined from the corresponding calibration curve.

### 2.6. Cryogenic Transmission Electron Microscopy

The morphology of GaPP-LCNPs following freeze-drying was imaged using Glacios 200 kV Cryo-TEM (Thermo Fisher Scientific). A total of 5 μL aliquot of GaPP-LCNPs was applied to 300-mesh copper grids that were glow discharged for 30 s. A mixture of liquid ethane/propane was used for sample vitrification, and the samples were kept at −180 °C during observation. Micrographs were recorded using a NANOSPRT15 camera (Thermo Fisher Scientific) operating a microscope at 120 kV under a bright field. All the reagents used were of analytical reagent grade to avoid any interference with imaging.

### 2.7. Small-Angle X-ray Scattering (SAXS)

The influence of trehalose on LCNP crystalline structure before and after freeze-drying was determined using small-angle X-ray scattering (SAXS). The measurements were conducted at the Australian Synchrotron (Melbourne, Australia) using a Bruker Nanostar system fitted with an X-ray source operating at 11 keV, 100 mA and Cu-Kα radiation with a wavelength of 1.27 Ȧ. The sample-to-detector length was set to cover the *q* range (0.05 to 0.4) relevant for these samples, and the scattering pattern was collected using a Pilatus 1M detector.

### 2.8. Illumination Setup

The illumination setup used for light activation was composed of a blue LED lamp emitting light at 405 nm (M405L4) [7]. Using an aspheric condenser lens (Ø1″, *f* = 16 mm, NA = 0.79, ARC: 350–700 nm) attached to the LED lamp with an SM1 Lens Tube, 1.00″, the light beam was collimated to illuminate 1 cm^2^ area. A T-Cube LED Driver (LEDD1B) was used to control the output power, which was monitored using a PM100USB power meter connected to a S302C thermal sensor head; all were purchased from Thorlabs (Newton, NJ, USA).

### 2.9. Determination of Singlet Oxygen Quantum Yield

The singlet oxygen quantum yield (${ɸ}_{\Delta }$) following photoactivation of GaPP-LCNPs was determined indirectly using uric acid as a probe [6,24]. Briefly, uric acid (0.12 µM) dissolved in 0.001% *v/v* Tween 20 was mixed with GaPP-LCNPs (1.2 µM) in a 1 mL quartz cuvette, followed by light activation at an output power of 20 mW/cm^2^. The declines in uric acid absorbance at 291 nm and GaPP-LCNPs at 405 nm were determined every 30 s for 5 min using an Evolution ^TM^ 201/220 UV-VIS spectrophotometer. The photodynamic activity was calculated using Equation (1) [6,24]:

(1) $$PA=\frac{{\Delta Abs}_{\left(UA\right)}\times 10^{5}}{E\times t\times PS}$$

where PA is the photodynamic activity of GaPP; ΔAbs _(UA)_ is the delta absorbance of uric acid before and after illumination multiplied by a correction factor (10^5^) for (t) time; E is the output power of blue light (mW/cm^2^); and PS is the absorbance of GaPP at an irradiation wavelength of 405 nm following illumination.

Following determination of the photodynamic activity, the singlet oxygen quantum yield was calculated using Equation (2) [6,24]:

(2) $${ɸ}_{\Delta }=\frac{{ɸ}_{\Delta }^{S}}{{ɣ}_{\Delta }^{S}}ɣ\Delta$$

where ${ɸ}_{\Delta }$ is the singlet oxygen quantum yield; ${ɸ}_{\Delta }^{S}$ is the singlet oxygen quantum yield of the standard photosensitizer, Rose Bengal = 0.79 [25]; ${ɣ}_{\Delta }^{S}$ is the photodynamic activity of Rose Bengal determined using Equation (1); and ɣ_Δ_ is the photodynamic activity of GaPP.

### 2.10. Determination of Antibacterial Activity

The minimum inhibitory concentration (MIC) of GaPP-LCNPs against *S. aureus* Xen 29 before lyophilization and after rehydration was determined using the broth microdilution method as previously described [26]. Briefly, a single colony of *S. aureus* Xen 29 from a freshly streaked agar plate was suspended in TSB overnight. The culture of Xen 29 in TSB broth was adjusted to 0.5 McFarland, followed by 1:100 dilution in cation-adjusted Muller–Hinton broth (CaMHB). Two-fold dilutions of GaPP-LCNPs and blank LCNPs were prepared in CaMHB and mixed with bacterial suspension in 96-well plates (5 technical replicates per plate), and one row containing nanoparticles in CaMHB without bacterial cells served as a blank for the turbidity measurement. MIC was identified as the minimum concentration that inhibited bacterial growth, where no visible bacterial growth or turbidity was detected.

## 3. Results and Discussion

### 3.1. Physicochemical Characteristics of LCNP Dispersion and Rehydrated Powder

**Figure 2:**
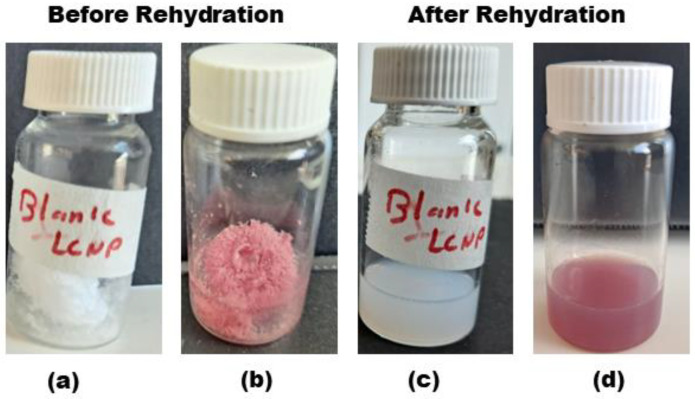
Illustrative images showing the physical appearance of blank LCNPs and GaPP-LCNP dry cakes cryoprotected with 2% *w/v* trehalose following freeze-drying (**a**,**b**), respectively, and the uniform dispersions (**c**,**d**) after rehydration.

Herein, we investigated the potential of trehalose in the concentration range between 0.5 and 5% *w*/*v* as a cryoprotectant to produce dispersible powder of LCNPs and GaPP-LCNPs. At lower concentrations (0.5% and 1% *w*/*v*), sticky masses of LCNPs were formed. However, at higher concentrations (2% and 5% *w*/*v*) of trehalose, we obtained dry porous dispersible cakes of LCNPs and GaPP-LCNPs (Figure 2a,b). Upon rehydration, all LCNP blank formulations (0.5–5 *w*/*v*%) formed milky white dispersions that were characteristic for LCNPs with no sign of aggregates. On the other hand, sticky masses of GaPP-LCNPs at 0.5% and 1% *w*/*v* of trehalose could not be fully dispersed, with GaPP aggregates remaining on the container’s wall (see Supplementary Figure S1). However, at higher trehalose concentrations, i.e., 2% and 5% *w/v*, the dry cakes were efficiently dispersed, forming uniform dispersions (Figure 2d), which implied trehalose conferred effective protection to GaPP-LCNPs at these concentrations that allowed the reformation of GaPP-LCNP dispersions.

**Figure 3:**
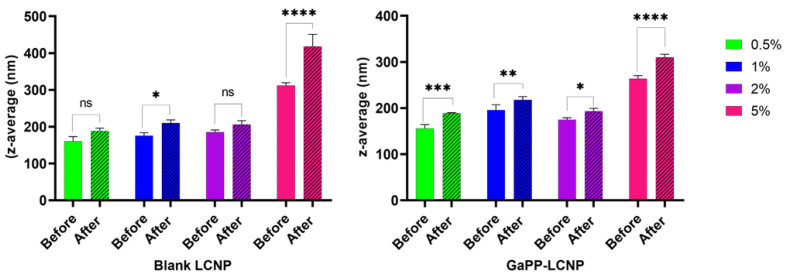
z-average diameter of blank LCNP and GaPP-LCNP dispersions containing trehalose in the concentration range between 0.5 and 5 *w*/*v*%. Date are presented as mean ± SD, n = 3; ns: non-significant; * *p* = 0.03, ** *p* = 0.006, *** *p* = 0.0001, and **** *p* < 0.0001. (Two-way ANOVA test followed by Sidak’s multiple comparison test).

The influence of incorporating trehalose on the hydrodynamic diameters of LCNPs with and without GaPP was investigated, and the diameters of the formulations were compared to the original dispersions without trehalose. In the concentration range between 0.5 and 2 *w*/*v*% trehalose, the nanoparticles had a z-average diameter < 200 nm with a polydispersity index (PDI) ≤ 0.2, similar to LCNPs prepared without trehalose [6,8]. However, at 5% *w/v*, the size of LCNPs increased to ~300 nm with a PDI ≥ 0.31 (Figure 3). This increase in size could be attributed to the entrapment of trehalose within the water channels of LCNPs.

The rehydrated LCNPs were larger in size compared to the dispersions before lyophilization. The increase in hydrodynamic diameter was more pronounced in the formulations containing 5% *w*/*v* trehalose (*p* < 0.0001), where the recorded z-average dimeters were 418 ± 33 nm and 312 ± 7 nm for LCNPs and GaPP-LCNPs, respectively, with a PDI ≥ 0.3. On the other hand, formulations containing 2% *w*/*v* trehalose had z-average diameters of 206 ± 10.5 nm and 193 ± 6.5 nm for LCNPs and GaPP-LCNPs, respectively, with a PDI ~ 0.2 (see Supplementary Table S1). The better size distribution and smaller hydrodynamic diameter of ~200 nm, in addition to the formation of dispersible cakes, prompted the use of 2% *w*/*v* trehalose as a cryoprotectant for GaPP-LCNPs in subsequent investigations.

**Figure 4:**
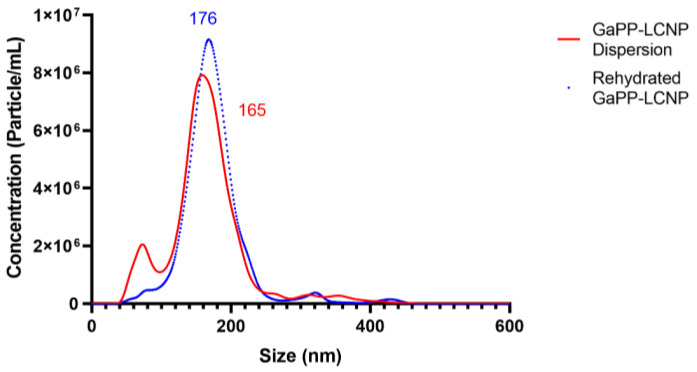
Mean particle diameter of GaPP-LCNPs with trehalose (2% *w*/*v*) before lyophilization (red) and after rehydration (blue). Data are presented as the average mean diameter obtained from NTA for 5 runs at 60 S each.

To further investigate the reproduction of LCNPs following rehydration, we determined LCNPs’ concentration (number of nanoparticles/mL) and particle diameter using nanoparticle tracking analysis (NTA). Akin to the hydrodynamic diameter data, NTA showed an increase in the particles’ diameter (Figure 4). However, no significant change in nanoparticle concentration was observed (Student’s *t*-test *p* = 0.4). This indicated that most nanoparticles were redispersed upon rehydration [27], as shown in Table 1.

The increase in diameter following rehydration can be attributed to the merging of smaller colloidal particles to form larger LCNPs in the reconstitution step. The particle-size distribution curve before lyophilization (red) shows a mean particle diameter of 165 nm and a small peak indicating the presence of nanoparticles with a dimeter lower than 50 nm. However, the small peak disappears in the blue curve representing reconstituted particles with a higher mean diameter of 176 nm, which indicates the formation of larger LCNPs.

### 3.2. Effect of Freeze-Drying on the Crystalline Structure of LCNPs

**Figure 5:**
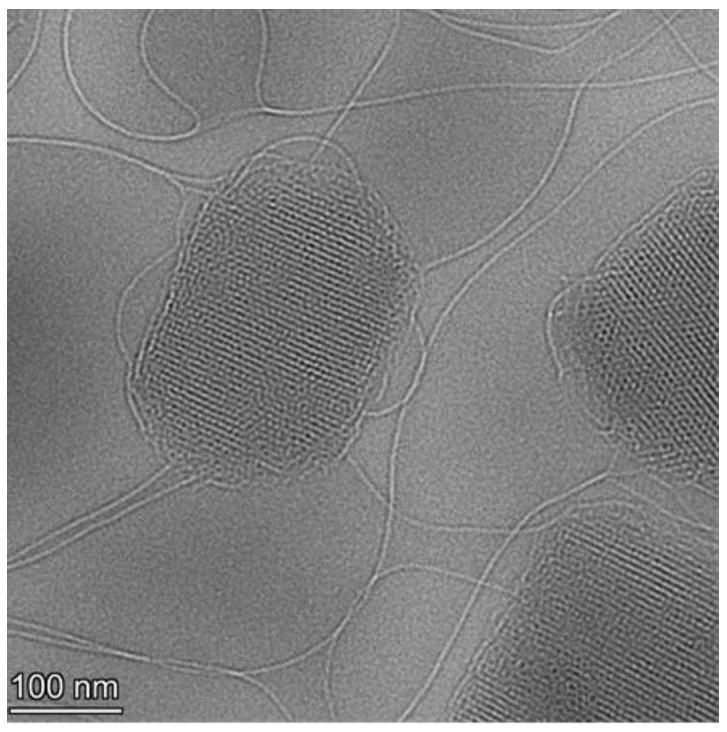
Cryo-TEM images of lyophilized GaPP-LCNPs following redispersion showing the cubic crystalline form of LCNPs with mesh-like lipid bilayer.

Following the investigation of the effect of lyophilization on LCNP particle diameter, we determined its effect on the crystalline structure of LCNPs. Firstly, the morphology of GaPP-LCNPs following rehydration was investigated using Cryo-TEM. The images indicate that GaPP-LCNPs maintained the crystalline mesh-like structure of LCNP lipid bilayer following rehydration (Figure 5).

**Figure 6:**
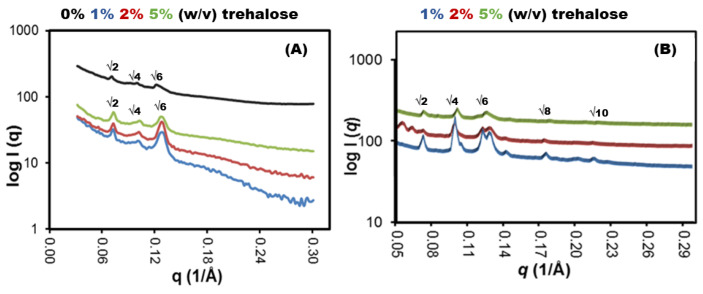
SAXS profiles of LCNPs at 25 °C. (**A**) LCNP before freeze-drying showing q peaks at √2, √4 and √6, indicative for Im3m phase. A value of 0% indicates LCNP without trehalose, while 1%, 2% and 5% *w/v* indicate different trehalose concentrations being tested. (**B**) Represents LCNP regaining the Im3m crystalline structure showing q peaks at √2, √4 and √6.

Moreover, we used SAXS to determine the influence of trehalose on LCNP crystalline structure. Before the addition of trehalose, LCNPs had an *Im3m* cubic structure at 25 °C, which agrees with previous reports [28,29,30]. There was no change in the crystalline structure upon the addition of trehalose at all tested concentrations (1, 2 and 5% *w*/*v*), where the SAXS profiles showed peaks at √2, √4, √6 and √8 that were indicative for primitive cubic *Im3m* phase [31], with a lattice parameter of 121.7 Å compared to 126.5 Å in the absence of sugar. The non-significant effect of trehalose on the internal structure of LCNPs was supported by the scattering peaks observed at the same *q*-values (Figure 6).

After redispersion in MQ water, LCNPs regained their *Im3m* crystalline form as demonstrated from the q peaks at √2, √4, √6 and √8, with a lattice parameter of 119 Å. The negligible change in lattice parameter and the reformation of the crystalline structure confirm the protective nature of trehalose during lyophilization. Furthermore, these results imply that trehalose did not occupy the space between the polar head groups of the monooleate lipid bilayer as previously reported with liposomes [19,32]; rather, it formed a glassy amorphous matrix around the lipid bilayer. In addition to the retained integrity of LCNPs, the reproduced nanoparticles efficiently entrapped GaPP molecules following the redispersion process, with GaPP entrapment efficiencies of 98.2 ± 3.2% and 99.2 ± 4.2% before and after lyophilization, respectively. It is noteworthy that the efficient entrapment of GaPP within LCNPs was observed when 2 and 5% *w*/*v* trehalose were used, while a reduction of >15% in the entrapment efficiency was reported at lower trehalose concentrations due to the incomplete redispersion of the formed sticky masses.

### 3.3. Effect of Lyophilization on the Antimicrobial and Photodynamic Activities of GaPP

The main aim of this study was to promote the stability and shelf-life of GaPP-LCNPs to advance their clinical application. Thus, it was fundamental to probe the activity of GaPP-LCNPs before and after lyophilization. Since GaPP is a metalloporphyrin complex, it can form molecular clusters inside the lipid bilayer through the magnetic properties of metal ions [33]. However, we previously demonstrated that through properly adjusting GaPP to lipid molar ratio, GaPP was uniformly distributed in its monomer form, which improved both the photodynamic and antimicrobial activities of GaPP [6,8]. Therefore, we quantified the singlet oxygen quantum yield (ɸ_Δ_) of GaPP-LCNPs using Equations (1) and (2). Interestingly, the calculated singlet oxygen value after the addition of 2% *w/v* trehalose was similar to the obtained value with the original formulation (PA = 105 ± 0.85 and ɸ_Δ_ = 0.72). These results confirm the suggested protective mechanism of trehalose by forming a protective matrix without disturbing the integrity of the lipid bilayer.

Following testing the photodynamic activity of GaPP-LCNPs, we investigated the effect of trehalose on the antimicrobial activity of GaPP as an iron mimetic agent. Trehalose did not change the antibacterial activity of GaPP against *S. aureus*; the MIC value of GaPP-LCNPs against *S. aureus* Xen 29 was similar to the original formulation (without trehalose) at 0.125 µg/mL, which was a significantly lower value compared to unformulated GaPP at 0.5 µg/mL. Moreover, rehydrated GaPP-LCNPs retained their antibacterial activity and inhibited bacterial growth at the same concentration (Table 2).

The better antibacterial activity of GaPP as an iron mimetic agent within LCNPs is attributed to the better solubility of GaPP molecules, which promotes their uptake via hemin receptors on *S. aureus* cell membranes [34], in addition to the curvature of the LCNP lipid bilayer that allows strong fusion abilities of LCNPs with bacterial barriers [35]. The success of rehydrated GaPP-LCNPs in retaining their antibacterial and photodynamic activities is a promising finding that will pave the way for future studies to investigate the shelf-life of LCNP formulations and test their antibacterial activity in more complex in vivo models, which will help scale up the manufacturing of GaPP-LCNPs and facilitate their utilization as a novel antimicrobial.

## 4. Conclusions

In this study, freeze-drying was used to produce a dry powder of GaPP-LCNPs. To the best of our knowledge, this is the first study to investigate the effect of lyophilization on the functionality of LCNPs loaded with a hydrophobic antimicrobial agent. This study demonstrated the ability of the loaded GaPP to retain its photodynamic and antimicrobial activities following freeze-drying and rehydration. Moreover, trehalose (2% *w*/*v*) was proven as an efficient cryoprotective agent for the formulation without altering the physicochemical characteristics. The rehydrated formulations were monodispersed with no significant change in LCNP concentration. Moreover, trehalose efficiently protected LCNPs without affecting the structure of the monooleate lipid bilayer. The efficient protective mechanism of trehalose preserved loaded GaPP with an entrapment efficiency of 99.2 ± 4.2% after rehydration. Most importantly, the functionality of GaPP-LCNPs as an antimicrobial agent and a photosensitizer was not changed after rehydration (ɸ_Δ_ = 0.72, MIC = 125 µg/mL). This study paves the way for future investigations on the shelf-life and storage conditions of LCNP dispersions to promote their application as a delivery system.

## Figures and Tables

**Table 1:** Comparison of mean particle diameter and nanoparticle concentration between LCNPs and GaPP-LCNPs before and after lyophilization.

	Original LCNPs		Reconstituted LCNPs	
Sample	Mean Diameter	Concentration of Nanoparticles/mL	Mean Diameter	Concentration of Nanoparticles/mL
Blank LCNPs	154 ± 3.8	7.8 × 10^8^ ± 1.1 × 10^8^	180 ± 5.1	7.5 × 10^8^ ± 3.6 × 10^7^
GaPP-LCNPs	165 ± 4.2	6.8 × 10^8^ ± 4.9 × 10^7^	176 ± 4.3	6.5 × 10^8^ ± 2.1 × 10^7^

**Table 2:** Minimum inhibitory concentrations of GaPP in LCNPs before and after lyophilization process compared to unformulated GaPP dissolved in 1% DMSO. The data present better antibacterial activity of GaPP in LCNPs with no affect for the addition of trehalose (n = 6).

Formulation	Unformulated GaPP	GaPP-LCNP (Original)	GaPP-LCNP (2% *w/v* Trehalose) Before Freeze-Drying	GaPP-LCNP (2% *w/v* Trehalose) After Rehydration
MIC (µg/mL)	0.5	0.125	0.125	0.125

## Supplementary Materials

The following supporting information can be downloaded at https://www.mdpi.com/article/10.3390/antibiotics12091405/s1, Figure S1: Illustrative images showing the physical appearance of GaPP-LCNP steaky cakes cryoprotected with 0.5% and 1% *w*/*v* trehalose following freeze-drying (a & b) respectively and the dispersions (c & d) after rehydration showing some steaky aggregates on the glass vial walls.; Table S1: Summary of z-average diameter and polydispersity index of LCNP and GaPP-LCNP before and after the lyophilization process.
