# Supplementary material for: Lyophilized Lipid Liquid Crystalline Nanoparticles as an Antimicrobial Delivery System
Source: Antibiotics (Basel). 2023 Sep 4;12(9):1405. doi: 10.3390/antibiotics12091405 (PMC10525386; doi:10.3390/antibiotics12091405)
Supplement: Supplementary file 1 [file antibiotics-12-01405-s001.zip › antibiotics-2586557-supplementary.pdf]

Supplementary materials

Lyophilized lipid liquid crystalline nanoparticles as an antimicrobial delivery system.

Muhammed Awad [1,2,3], Timothy J. Barnes [1,] and Clive A. Prestidge

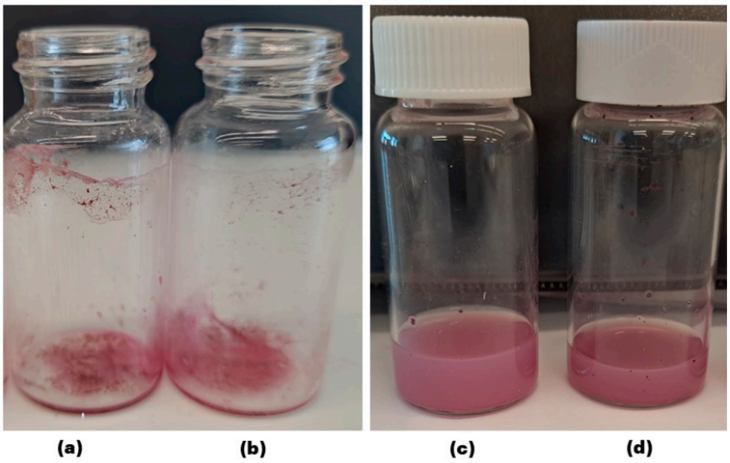

Figure S1. Illustrative images showing the physical appearance of GaPP-LCNP steaky cakes cryoprotected with 0.5% and 1% w/v trehalose following freeze-drying (a& b) respectively and the dispersions (c & d) after rehydration showing some steaky aggregates on the glass vial walls.

Table S1. Summary of z-average diameter and polydispersity index of LCNP and GaPP-LCNP before and after the lyophilization process.

	LCNP before freeze drying		Reconstituted LCNP	
Sample	z- average diameter (nm)	polydispersity index (PDI)	z- average diameter (nm)	polydispersity index (PDI)
Blank LCNP 0.5% trehalose	160 ± 13	0.28 ± 0.02	189 ± 7.1	0.23 ± 0.02
Blank LCNP 1% trehalose	175 ± 8.6	0.22 ± 0.04	210 ± 9.1	0.28 ± 0.03
Blank LCNP 2% trehalose	185 ± 6	0.21 ± 0.04	206 ± 10.5	0.23 ± 0.05
Blank LCNP 5% trehalose	312 ± 7	0.18 ± 0.14	418 ± 33	0.36 ± 0.03
GaPP-LCNP 0.5% trehalose	156 ± 8.5	0.19 ± 0.03	189 ± 1.3	0.23 ± 0.02
GaPP-LCNP 1% trehalose	196 ± 11.4	0.21 ± 0.04	218 ± 6.8	0.34 ± 0.05
GaPP-LCNP 2% trehalose	175 ± 4	0.20 ± 0.04	193 ± 6.5	0.15 ± 0.02
GaPP-LCNP 5% trehalose	264 ± 7	0.32 ± 0.05	312 ± 7	0.35 ± 0.02
